# Emergence of multilateral proto-institutions in global health and new approaches to governance: analysis using path dependency and institutional theory

**DOI:** 10.1186/1744-8603-9-18

**Published:** 2013-05-10

**Authors:** Eduardo J Gómez, Rifat Atun

**Affiliations:** 1 Department of Public Policy & Administration, Rutgers University, 401 Cooper Street, Camden, NJ 08102, US; 2 Imperial College Business School and the Faculty of Medicine, Imperial College London, South Kensington Campus, London SW7 2AZ, UK; 3 Harvard School of Public Health, Harvard University, 677 Huntington Avenue, Boston, MA 02115, US

## Abstract

The role of multilateral donor agencies in global health is a new area of research, with limited research on how these agencies differ in terms of their governance arrangements, especially in relation to transparency, inclusiveness, accountability, and responsiveness to civil society. We argue that historical analysis of the origins of these agencies and their coalition formation processes can help to explain these differences. We propose an analytical approach that links the theoretical literature discussing institutional origins to path dependency and institutional theory relating to proto institutions in order to illustrate the differences in coalition formation processes that shape governance within four multilateral agencies involved in global health. We find that two new multilateral donor agencies that were created by a diverse coalition of state and non-state actors, such as the Global Fund to Fight AIDS, Tuberculosis and Malaria and GAVI, what we call proto-institutions, were more adaptive in strengthening their governance processes. This contrasts with two well-established multilateral donor agencies, such as the World Bank and the Asian Development Bank, what we call Bretton Woods (BW) institutions, which were created by nation states alone; and hence, have different origins and consequently different path dependent processes.

## Introduction

The governance and responsiveness of multilateral donor agencies working in the area of global health is an emerging area of research. Since 2000, coinciding with the creation of Millennium Development Goals and increased overseas development assistance for health, the importance of these agencies in combating diseases and poverty has increased. Yet, there is limited research exploring how these agencies have organized themselves to improve their effectiveness and to meet the health related Millennium Development Goals.

Earlier studies indicate that multilateral funders, such as the World Bank, the Global Fund to Fight AIDS, Tuberculosis and Malaria (Global Fund), and the Global Alliance for Vaccines Initiative (GAVI) have attempted to reform their governance arrangements in order to improve transparency, accountability, and responsiveness to countries and citizens’ healthcare needs in response to pressures from governments and non-governmental organizations (NGOs) [1-5]. However, extant research also highlights wide variation in the outcomes of governance reforms undertaken by multilateral donor agencies, with studies suggesting that the governing boards of these agencies have failed to improve transparency in their decision-making, to consistently engage NGOs and other stakeholders in decision-making, and to expand broad representation and voting influence within governing boards; suggesting obfuscation and use of ‘immunity’ privileges have hampered change efforts and to hold individuals within these agencies accountable for wrongdoing, underperformance or mismanagement [6]. Hence, it is not clear whether multilateral donors have increased transparency, representation and inclusiveness, accountability and responsiveness to country and civil societal healthcare needs, or what we refer to in this study as *strengthened governance processes*, which is the primary outcome of interest in this paper.^a^

Specifically, we find that multilateral donor agencies, which have their origins in Bretton Woods (BW) Institutions, and United Nations agencies which have evolved with the Bretton Woods philosophy, have struggled to fully achieve strengthened governance processes. Agencies such as the International Monetary Fund and the World Bank emerged following the meetings in 1944 of many nation states in Bretton Woods, New Hampshire, USA. Broadly, BW Institutions also include multilateral agencies, such as the Asian Development Bank (ADB), the Inter-American Development Bank (IDB), and the African Development Bank, that have emerged subsequently, but share distinct similarities with the organizational structure and goals of the World Bank. Another common feature shared by the BW institutions is that they were created by influential and wealthy nation states, such as the United States, the United Kingdom, and Japan.

Since 2000, new multilateral agencies in global health have emerged with features that differ from BW institutions. These new agencies have not followed the typical evolutionary path of BW Institutions. Instead, the ideas guiding their creation have emanated not from political elites of nation states, as was the case with BW institutions, but from the interests and strategies of a range of non-state actors. Following Lawrence et al. [7], we refer to these agencies as *proto-institutions*, where the rules of governance within agencies were created by extensive collaboration and coordination between civil society groups. Rules of governance within agencies reflect their interests, in turn helping ensure strengthened governance processes with broadened accountability to multiple stakeholders.

Two examples of these proto-institutions include the Global Fund and GAVI, which were established in 2000 and 2002, respectively. An important distinguishing feature of these proto-institutions is that they appear committed to implementing policies aimed at increasing their decision-making transparency, their representation of a broad range of stakeholders that include civil society (i.e., individuals in need of healthcare, individuals and communities affected by communicable and non-communicable diseases, as well as a range of non-governmental organizations that advocate for their needs), private foundations, and the private sector in their decision-making processes.

The emergence of multilateral proto-institutions warrants research into explaining why and how they have diverged from the Bretton Woods institutions. Indeed our key research question in this article is the following: what accounts for differences in strengthened governance processes between the newer proto-institutions as compared with multilateral agencies that are Bretton Woods institutions? We argue that understanding these differences requires the application of theoretical frameworks capturing these agencies’ origins and policy evolution. Our research, therefore, breaks new ground because to our knowledge, it is the first attempt to apply and use institutional theory to explain differences in outcomes between multilateral donor agencies in global health.

In this article, in contrast to studies which have explored the importance of endogenous organizational interests for achieving these governance outcomes within multilateral donor agencies, often kindled by well organized, pro-active civic mobilization and pressures [1-3,5,6,8,9], we draw on research on institutional origins, proto-institutions, and path dependency theory to explain the creation and implementation of strengthened governance processes within multilateral donor agencies. While institutional origins and proto-institutional theory help to explain the initiating political actors, interests, and coalitional strategies involved in creating multilateral donor agencies, path dependency theory helps to explain how and why actors within these agencies behave the way they do *after* they have been created, e.g., why agency presidents and governing board members repeatedly were unable to change ineffective governance processes, rules and policies.

We propose a testable hypothesis: that the emergence of strengthened governance process depends on the types of actors, interests, and coalitions involved in the historic formation of multilateral donor agencies in global health. More specifically, our proposition is that multilateral agencies created by a single or small group of influential, predominant, industrialized nations devising coalitions of reform and have origins rooted in Bretton Woods ethos have been less successful in achieving strengthened governance processes. Conversely, multilateral donor agencies created by the strong involvement of civil society, with the support of other non-state actors, such as the private sector, will lead to new interests and reform coalitions creating agencies that are more successful in achieving strengthened governance processes. But what our argument ^b^ seems to suggest, and what the relevant literature has essentially ignored, is that multilateral agency performance in these strengthened governance processes is historically predetermined, with little prospect for concrete reforms within BW Institutions, that is, the willingness to implement and more importantly, remain committed to and enforce policies that strengthen these processes. While all four agencies studied were successful in implementing new governance policies, the Global Fund and GAVI demonstrated a persistent commitment to enforcing governance policies, such as the provision of decision-making information for increased transparency, accountability to civil society by including civil society and other non-state actors in multiple governance levels, from governing boards to board committees, as well as in-country coordination mechanisms, frequently meeting with civil society, and ensuring that civil society views and interests of other key stakeholders were consistently included in policymaking processes.^c^

## Methods

Our study drew on analysis of the published literature and publicly available data from journal articles, books, agency web cites and publication, media, and research reports.

We compared the World Bank, ADB, Global Fund, and GAVI. There were, inter alia, three main reasons for purposively selecting these cases for comparison. First, we were very familiar with these cases, being familiar with their reform efforts, having conducted and published research on them, as well working within them, e.g., the World Bank and the Global Fund; moreover, instead of comparing other UN regional banks and global funding initiatives, writing about cases that we were familiar with allowed us to provide rich causal detail and case study illustrations, an approach that helps to illuminate the potential utility of a proposed theoretical approach [10,11].

Second, we selected these four cases because we had a strong expectation of the differences in outcomes of reform processes and the strengthened governance processes between them; thus allowing us to provide alternative explanations for the factors leading to these governance outcomes. Purposive selection of cases based on known outcomes is justifiable when scholars strive to provide new theoretical insights into the causes leading to these known outcomes [10,12].

And third, with respect to the Global Fund and GAVI, we choose these cases because they were the most established agencies of their kind (i.e., non-UN multilateral financiers of global health) with global reach. We were, hence, able to obtain information about their governance structure and policy performance over a substantial period of time. Other non-UN multilateral financiers providing drugs and funding for diagnostics were created at later points in time, such as UNITAID in 2006. There exist other multi-lateral agencies, public-private partnerships, and philanthropic initiatives, which we could have examined. However, in order to provide a focused, in-depth discussion, and for the aforementioned reasons, we restricted our analysis to the World Bank, ADB, Global Fund, and GAVI.

### Approaches to multilateral agency governance

The role of multilateral agencies in global health and their transformation to increase transparency, representation, accountability and responsiveness to country and civil societal health needs is a new area of scholarly research. Civil societal health needs represent individuals’ needs for access to medication, healthcare treatment, as well as information for awareness and prevention. These individuals often include persons who are at high risk of illness, but who cannot afford medications or healthcare services. Country health needs refer to governmental and societal needs in providing essential medicines, funding for prevention, treatment, as well as strengthening health systems and the delivery of medications.

Increasingly, a number of scholars have argued that multilateral health agencies should be more committed to ensuring the representation and needs of civil society and not just those of nation-state representatives [13-16]. This argument is based on the premise that while nation-state representatives may embody the interests of civil society they do not necessarily convey societal needs, as they may not have relevant and timely information about these societal needs or do not have incentives to learn about them, hence, leading to differences in policy priorities between national governments and civil society [15]. To avoid these gaps in societal needs and polices, there have been calls for direct representation by civil society and those affected by diseases within governing mechanisms of agencies involved in global health. Such representation, it is argued, would enable individuals affected by diseases to share their experiences with policymakers and help devise policies that are responsive to societal needs [13,15]. However, does representation on a governing board guarantee its responsiveness and accountability to civil and broader society? While representation on boards does not guarantee this outcome, increased presence of civil society activists and NGOs does increase the likelihood that their views will be heard. While the founding charters of *proto institutions* in global health, such as the Global Fund and GAVI, express commitments to civic representation and involvement in decision-making, compelling their boards to pay attention to the needs of civil society, Bretton Woods institutions, such as the World Bank and the ADB, do not have civil society representation on their boards with less possibility for civil society views to be heard [2,3,6], an issue we explore in greater detail.

The scholarly literature has often claimed that efforts to strengthen governance processes are the product of individual interests among agency presidents and governing boards, to enhance organizational effectiveness in policy-making [1-3,5,6,8,9]. In contrast, others have argued that the well-organized pressure from civil society and transnational activist groups have influenced these transformations. These groups, it is argued, have effectively use the media to disseminate information on the transparency of multilateral agencies, and actively report instances of corruption and succeed in pressuring multilateral agencies into pursuing these outcomes [5].

However, there are limitations in this body of scholarly research. First, the focus of the research and the literature is on the reform of governance policies; but this tells us very little. For even if board members agree to new policies, there is no guarantee that these policies will be enforced. It is therefore necessary to understand *why* governing boards refrain from enforcing policies they have developed. And moreover, what are the historical and contextual factors that motivate the implementation of governance policies? The aforementioned literature is silent on these issues.

Second, earlier research has relied on the formal design of institutions, their interests, and outcomes. In accordance with the historical institutionalism literature [17], this approach begins with the assumption that the nature and design of governance mechanisms within agencies, such as governing board autonomy, technical capacity and cooperation with external members, determine the willingness and capacity of an agency to strengthen governance processes. Yet the impact of these governance mechanisms is questionable when there is little evidence suggesting that governing board members effectively adhere to and implement governance procedures. Hence, we agree with those positing that the rational design of institutions often fails to generate predictable policy outcomes [18,19].

Third, extant studies have not adequately considered the comparative historical origins of multilateral donor agencies and hence, do not explain why and how governing boards are designed the way they are, the actors and interests that created them, and how and to what extent the institutionalization of these interests remains and shapes the motivation and commitment of subsequent agency leaders. Furthermore, extant research has largely overlooked the evolutionary role of non-state actors, such as civil society, and their interaction with government officials during the formation of institutions. Since the mid-20^th^ century, while many civic organizations and social health movements have worked to increase awareness and knowledge on diseases affecting vulnerable populations, the role and influence of social health movements in global health increased in the 1990s, when these groups worked with others to establish novel international agencies, e.g., proto-institutions, and new international policy norms of access to medicine and treatment as a human right [20].

With respect to the creation of multilateral agencies, we uphold Pierson’s [21] notion that it is not “what” happens, but “when” it happens that is important: that is, early interests, coalitions, and institutional designs establish self-reinforcing patterns that sustain themselves over time, patterns that become difficult to reverse, regardless of the introduction of policy innovations and external pressures to change them [21]. Therefore, any explanation of the capacity of multilateral agencies to strengthen governance processes requires a historical analysis of their formation.

In this article, we combine research pertaining to institutional origins and *proto-institutions*, approaches that have examined historical roots, coalitions, and the politics leading to the formation of institutions [17,18,22-25], with research on path dependency [21,26,27], in order to reveal a deeper, more nuanced picture of the differences among multilateral donors in strengthening their governance processes.

Two areas of research, which examine the origins of institutions and their capacity to better explain and predict subsequent institutional and policy behavior, provide the analytical foundation for our study. First, research by Waldner [18] questions the efficacy of historical institutionalism [28] in its ability to describe and predict policy outcomes. This observation is based on the premise that specific types of political institutions often fail to generate the types of policies that are expected of them given their institutional designs, such as a concentration of power within the executive (viz., the presidency) and the expectation that this concentration of power facilitates economic policy-making. To avoid empirical error, Waldner [18] suggests treating national elite preferences, conflicts, and coalitions as variables *explaining the rise* of institutions as our outcome of interest. In so doing, he claims that one can better describe and therefore differentiate between similar types of institutions, e.g., authoritarian regimes. Other scholars have adopted a similar approach to Waldner [23,24,29]. For example, Donor, Ritchie, and Slater [22] maintain that the key to understanding variation in economic bureaucratic performance is not by treating formal bureaucratic design as causal variables, but as outcomes of concern, with national elite interests and coalitional survival strategies as key causes.

The second area of research that has provided analytic underpinnings of our study examines the formation of proto-institutions. Here, proto-institutions represent binding formal and/or informal practices and rules of governance within agencies and/or political institutions, as well policy innovations, as outcomes to be explained. These proto-institutions are the product of collaborations between non-governmental organizations. The extent of what Lawrence et al. [7] characterize as *involvement* and *embeddedness* in relationships between NGOs determines the likelihood of the emergence of proto-institutions. For example, limited interaction and sharing of information between only two NGOs, i.e., *involvement*, leads to a low likelihood of proto-institutions emerging, whereas extensive collaborations between multiple NGOs, as well as the flow of information beyond the initial collaborating parties, i.e., *embeddedness*, facilitates the diffusion of new ideas and rules beyond these negotiations. Embeddedness, in turn, leads to the formation of proto-institutions that are self-reinforcing through mechanisms of rewards and sanctions, thus taking on path dependent qualities.

**Figure 1:**
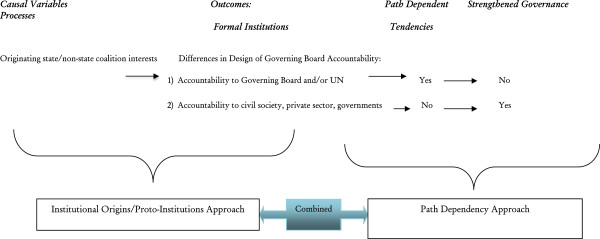
Protoinstitutional formation processes.

As Figure 1 illustrates, the underlying goal of an analysis that combines institutional origins and proto-institutions approaches is to understand the *formation* of different types of institutions. This formation is based on differences in institutions’ originating coalitions interests, which are treated as causal variables. Our outcome of interest is the presence of governing boards that are successful in achieving strengthened governance processes, that is, decision-making transparency, representativeness and inclusiveness, accountability, and responsiveness to country and civil societal needs.

We build on the literature on institutional origins and proto-institutions [18,19] to describe and differentiate between different types of multilateral donors in global health, such as the World Bank, ADB, the Global Fund, and GAVI, by analysing in these institutions originating elite interests, coalitions, and reform strategies. Equipped with better analysis of the origins of these multilateral donors, we can more accurately describe, anticipate, and understand their strengthened governance processes and, consequently, their likely adaptation to country and civil societal needs.

Combining the institutional origins and proto-institutions theoretical frameworks *with* path dependency, provides still deeper insights. As Figure 1 illustrates, this combination is achieved through a temporal, sequential analytical approach to explain causal processes [21]: that is, an approach where different theoretical frameworks and causal mechanisms are sequentially linked to each other and used to explain different phases of the institutional and policy development process [21]. As shown in Figure 1, during the initial period of institution formation, the institutional origins and proto-institutional literature applies, while the path dependency literature holds true at a later point in time, i.e., by explaining reform processes *after* these institutions and policies have emerged.

The complimentary aspects of path dependency we emphasize, namely *power* and *increasing returns,* are self-reinforcing mechanisms of institutional reproduction [21]. *Power* mechanisms determine the concentration of resources with a particular individual or group of individuals [27,30]. When high concentration of resources combines with political will to maintain the status quo, leaders will not have incentives to transform institutions as they will continuously benefit from controlling and manipulating them, even if they are aware of the inefficiencies of the status quo, which further underscores how firmly committed they are to using resources for personal gain [27,30]. With regard to *increasing returns*, a high level of initial financial and technical investments into a particular institutional design locks an institution onto a particular path, even when subsequent inefficiencies emerge [19,31]. This locking onto a path occurs, as over time the costs of reforming the institution greatly outweigh the benefits [21].

We also emphasize the challenges of institutional and policy-related *learning.* These are challenges where actors’ acquired knowledge of organizational design and policy, acquired either through instruction or through inheritance of information, provides deep familiarity and comfort with the organization and policy, and thus creates incentives to sustain existing design and policies [21,31]. Knowing the detailed intricacies of a bureaucratic/policy design increases the transaction costs of reform, such as the time and money needed to learn new approaches [21,31]. And according to the self-reinforcing mechanism of *learning,* once individuals have learned about a particular institution/policy and have seen its repeated effectiveness, popularity, and support, they assign a great deal of legitimacy and decide to safeguard it, despite its known inefficiencies [26].

Our combined analytical perspective, therefore, provides for two elements: First, by focusing on the origins of institutions and analysing processes that lead to the establishment of a proto-institution, we provide a more-nuanced description of the *types* of multilateral donor agencies present in global health. Second, equipped with a more nuanced description of multilateral donor agencies, we use path dependency literature to better understand the causal nature of policy reform processes within agencies and their ability to engage in strengthened governance processes*.*

Based on our proposed analytical framework, we hypothesize that the ability of multilateral donor agencies to strengthen governance process is first shaped by the types of constituencies their governing boards confront. As Figure 1 illustrates, accountability of multilateral donor agencies to governing institutions such as the United Nations with entrenched nation-state interests, or to a small group of influential nation-states, leads to slow moving, self-reinforcing path dependent tendencies. Conversely, when multilateral agencies are accountable to a wide range of stakeholders involved in global health, such as civil society, the private sector, philanthropists and NGOs, there is greater flexibility in policy experimentation and adaptation in order to strengthen governance processes and improve responsiveness to healthcare needs (Table 1).

**Table 1:** Multilateral donor agency governance arrangements and outcomes

	**Governing board membership**	**Accountability constituents**	**Strengthened governance processes**
**Bretton Woods Institutions**	Nation States	Horizontal (within agency) and vertical (to civil society)	Partial and/or weak
**Non-Bretton Woods Institutions**	- Nation States	Vertical (to civil society, private sector, and nation states)	High
	- Civil society		
	- Private sector		

### The World Bank

Originally called the International Bank for Reconstruction and Development (IBRD), the World Bank Group emerged following the international conference held in July 1944 in Bretton Woods, New Hampshire, USA. At this conference, 108 delegates from several nation-states discussed the creation of an international bank that would help European nations reconstruct their post-war economies and would provide financial and technical assistance to developing nations [4,32,33].

However, the main focus of the conference was not to create an international development bank, but instead an International Monetary Fund, which would bring about international cooperation and monetary stability, leading to, as some scholars have argued, a political coalition that was narrow, fragile, lacking focus, and based on consensus [4,32,33].

The initial idea of creating an international reconstruction and development bank was put forward by the United States (US) in 1943, by a Mr. Harry White, who initially approached and worked closely with delegates from the United Kingdom (UK), namely the Treasury Secretary John Maynard Keynes, who initially hesitated to adopt the idea, given both nations’ differences in international economic views and policies [4,32,33]. Prior to Bretton Woods, at a meeting held in Atlantic City, New Jersey, the US and the UK agreed to put forth the proposal at Bretton Woods. However, at Bretton Woods, their proposal for the bank received scant attention, while kindling debate and a lack of consensus not only over the name of the bank but more importantly, its focus on reconstruction (which the Europeans desired) versus development (which Latin Americans desired). Characterized by many as an “afterthought” at Bretton Woods, delegates from the US, UK, and developing nations agreed that the proposed bank would focus on both reconstruction and development [34]. However, the nature of the debates and the lack of attention given to the bank’s purpose created a lack of clear and coherent consensus and commitment [33,35].

Despite a lack of strong consensus and purpose, several conditions facilitated the emergence of the US as the dominant coalitional partner in crafting the Bank’s Articles of Agreement and governance structure: first, the high level of financial prowess of the US and the size of its initial investment, estimated at US$10 billion [32]; second, US stewardship in creating the idea of an international bank [33]; and third, the US’ geopolitical and economic influence [33]. These factors enabled the US Department of State and Treasury to take the lead in drafting the core elements of the Articles of Agreement [32,33]. Moreover, in exchange for a loan from the US, the UK permitted the US to draft the Articles of Agreement [32]. With this authority the US created Articles that provided for high level of US influence and power, such as becoming an initial major stock holder of the Bank, positioning the Bank on US soil (thus allowing for easier oversight and influence from the US President and Congress), as well as establishing an informal agreement that the Bank President would be an American citizen [4,32,33].

The US also drafted Articles that secured its interests in the Bank’s governance structure, guaranteeing the US a high proportion of the voting shares (through investment), voting power (which is a product of these voting shares), as well as secure and complete representation [33]. The Articles of Agreement ensured that the largest quota shareholders of the Bank, namely the US, UK, Japan, and France, made up the largest voting block and representation on the Board of Governors [4,33]. The smaller states, which possessed a correspondingly smaller share of investment and quota shares, had less voting influence [4,33]. The Articles also conferred upon the Bank’s Governing Board complete autonomy in policy-making, without accountability to the United Nations, the US Congress, or any other governing body [32], essentially making the Governing Board accountable to itself and the Board representatives of the nation-states that invested in the Bank [6]. The Articles were largely silent on the issue of ultimate accountability: that is, there was no clear indication as to whom the Board of Governors and the Executive Directors would be accountable to [32]. This lack of clarity on accountability ensured the autonomy and influence of large shareholders, but hindered the influence of stakeholders in national governments, civil society, and the private sector in the policy-making process. The initial elite interests and coalitions emerging at Bretton Woods led to the creation of an Articles of Agreement that hampered the World Bank’s equitable representation and broad accountability to civil society and other actors.

During the 1980s, civil society activists and NGOs who opposed structural adjustment policies espoused by the World Bank [36] also organized campaigns to pressurize the World Bank to increase its accountability and transparency [2]. In response the World Bank began to introduce policies that increased the governing board’s transparency, accountability, and partnership with civil society. For example, in 1980, the World Bank created the “NGO-World Bank Committee,” which was made up of NGOs and Bank staff [2,9]. And in 1995, President James Wolfenson authorized the creation of an “NGO Liaison Officer” [2].

Beginning in the early-1990s the Bank’s Governing Board also created Public Information Centers, which distributed information on projects and policy analysis [1]. By 2008, the World Bank Board had created more than 100 Public Information Centers and 200 additional information access points [1]. In 1993, in an effort to increase accountability for its policy actions, the World Bank Board agreed to the creation of the Inspection Panel [9,37]. Through this Panel, civic groups believing to be directly and negatively affected by World Bank policies were able to complain and request investigations into matters affecting them [9,37]. The Panel was invested with the authority to request Executive Directors to look into matters warranting detailed investigation [9,37].

In 1999, the World Bank further extended its commitment to stakeholder accountability and transparency by creating the Compliance Advisor/Ombudsman Office (CAO) [1]. The CAO works directly with civil societal groups that might be affected by the Bank’s International Financial Corporation (IFC) and Multilateral Investment Guarantee Agency’s (MIGA) procedures. The CAO reports to the World Bank President and is strictly accountable to that office [1].

Additionally, in an effort to further increase transparency and accountability to civil society and other stakeholders, in December 2009 the Governing Board of the World Bank established a new policy of increasing access to information, in order to increase stakeholder ownership and influence over the financing operations of the World Bank [1]. This policy required all financial transactions between the World Bank and lenders, as well as documents for Executive Directors and the Board of Governors, to be made available *before* Board meetings and to prior to decisions and policies. The policy was shaped by five major principles: (i) Maximizing access to information, (ii) Clear exceptions (such as denying access to information, should the information’s disclosure potentially harm “well-defined interests”), (iii) Safeguarding the deliberative process, (iv) Clear disclosure procedures, and (v) Having the right to appeal decisions over access to information. The overall policy was intended not only to increase access to information, but also to ensure more equitable and fair access to this information by stakeholders and governments [1].

While the World Bank has been lauded for recent efforts to become more transparent, emerging as one of first multilateral agencies to commit itself to greater transparency and broad accountability [38], a number of researchers have criticized the speed by which these policies have been implemented, the extent of engagement with NGO representatives and the limited incorporation of NGO recommendations into decision-making processes [2,8]. By far the biggest challenge to the World Bank, Ebrahim and Herz [2] argue, has been the consistency of information, transparency, and accountability from the Governing Board to civil society, a view shared by Jenkins [1].

In spite of visible efforts to enact policies and develop initiatives, why has it been difficult for the World Bank to consistently and vigorously implement actions to achieve greater transparency and accountability? We argue that the originating political elite interests and coalitions that have established the World Bank’s accountability structure created several path dependent processes that have hindered the World Bank to effectively implement policies for strengthened governance processes. First, in line with *increasing returns* theory [21], the US’ initial investment of US$10 billion, its purchase of an initial 20% share of the Bank’s stock, as well as the President and Congress’ selection of a Bank President and the US government’s drafting of the Articles of Agreement led to a high level of US initial investment into the World Bank [32]. This initial investment motivated subsequent World Bank Presidents, Governing and Executive Board Directors, as well as a supportive Congress [39], to sustain the governance arrangements. Indeed, Prah Ruger [40] maintains that throughout the 1970s the Governing Board was unable to respond to repeated demands from nation states and civil society for greater accountability and responsiveness, especially for health and other social welfare needs. Health policy became a priority when the World Bank President, Robert McNamara (President in 1968-81), asked his staff to conduct studies on the importance of health in development [40]. His actions were not prompted by government and civil society pressures; and even when health policy emerged as a priority under President McNamara, it led to loans and technical advice to developing nations [40], but not accountability and civil societal influence in health policy decision-making [2]. Thus, in line with *increasing returns* theory, an initial high investment into an existing institution generated incentives to perpetuate the institution’s design, purpose, and influence, notwithstanding its acknowledged policy gaps and repeated calls for reform.

Second, the path dependent mechanism of *power*[27,30], emerging from the original coalitional arrangements, facilitated in turn the Governing Board’s influence. Power in path dependent processes emerges when actors (in this case, dominant shareholders) posses a high degree of financial and political resources, which facilitate their ability to make decisions and resist new institutional and policy designs [27,30]. Following the Bretton Woods Conference, major shareholders were able to concentrate their power financially and become suppliers of private sector funds through banks in major financial centers [34].

Furthermore, the World Bank’s informal institutional influence, mainly through bilateral agreements and loans to powerful allies, such as France and Japan, further augmented its concentrated power and influence [4,32] through the World Bank president and the president’s representative on the Governing Board [39].

A further path dependent factor emerging from the World Bank’s originating coalitional politics is institutional and policy *learning* and *legitimacy*[21,26,31]. As noted earlier, these self-reinforcing mechanisms emerge when actors within organizations master a particular bureaucratic and policy framework, either through sharing information or through instructions [21,26,31]. Such bureaucratic arrangements and policy frameworks achieve critical mass as a result of their popularity and support among key leaders, with a great deal of legitimacy assigned by staff to a particular bureaucratic and policy framework. Eventually, these actors resist policy change, even with flawed or inefficient policies, because of the learning investment placed in the current design and prospect of a similar type of investment any new design would necessitate [21,26,31]. In the case of the World Bank, scholars note that government representatives involved in the creation of the Bank at Breton Woods recognized the US, UK, and other advanced industrialized nations’ commitment to the Bank’s Articles of Agreement [32], with the emergence of an organization highly committed to its mode of governance and accountability to stakeholders, creating a mode of leadership and direction that supported the president’s policy interest and aspirations [4,32].

Consequently, despite periodic demands for greater accountability, and despite gradual policy movements in this direction, it has been difficult to substantially reform the World Bank’s approach to transparency and accountability to external stakeholders to be more responsive to country needs and more importantly, the needs of civil society.

### Asian Development Bank

The origins of the Asian Development Bank date back to 1966. The ADB closely resembled the mission and organizational structure of the World Bank. It was created as a multilateral agency comprised of a Governing Board of investors and Executive Directors managing policy implementation. The overall mission of the ADB was to provide economic and technical assistance to the Asian region, mainly in the form of loans, to help develop infrastructure and implement structural adjustment policies; it was also created to help implement and manage free-market systems [41,42]. Initially 31 member countries constituted the ADB. The agency now has 67 member states [42,43]. By the late 1990s, the ADB became more involved in addressing poverty [42], joining the World Bank and other multilateral agencies to forge partnerships with governments and civil society to achieve this objective [44].

The political origins of the ADB were marked with disagreements among constituent countries and a lack of coalitional consensus. While the idea of creating a regional bank in Asia emerged as early as 1954, based on discussions from members of the Economic Commission for Asia and the Far East (ECAFE), the idea was initially resisted by Japan and the US [45]. Facing ongoing stalemate, in 1963, at the 19^th^ session of ECAFE, the chairman of ECAFE was able to build consensus and secure agreement between Japan, the US and other countries to form the ADB [45]. With the specter of communism expanding over Southeast Asia, especially Vietnam, the US viewed a strong regional bank helping countries to develop and break away from communist influence.

As two of the most influential countries investing in the ADB’s formation, Japan and the US sought to establish a charter constitution reflecting their policy interests as well as the views of other non-Asian industrialized nations, often dominating those of lesser-developed countries, as the former controlled two thirds of the voting power through their investments in the ADB [45]. Furthermore, through an informal “gentleman’s agreement,” the US agreed that Japan should appoint an ADB President, with a five-year term, to chair the Governing Board [41,45], which some scholars explain entailed an emphasis on infrastructural development and free market reforms fostered by Japan and the US [45].

By the 1970s and 1980s, the ADB Governing Board was accountable mainly to three constituents: the nation states represented on the Governing Board, the President of the ADB (Japan), and board representatives from Japan and the US. While, in theory, the Governing Board was accountable to citizens of the member nations, in reality such direct lines of representation did not materialize [41,42]. Initially, the ADB did not establish a close relationship with civil society and, therefore, it was not accountable to them [41,42]. In several instances, for example, the ADB often negotiated with government officials for the financing of specific policies without incorporating the views of civil society [41]. In 2001, for example, in Thailand, the villagers from Klong Dan village (located near Bangkok) complained that they had not been consulted prior to the ADB and government’s approved funding of a wastewater treatment facility in Klong Dan, requesting reassessment of the project and for the ADB board to increase its accountability and interests to their needs in the future [46].

In 1999 a *Special Evaluation Study* conducted by the ADB discovered a shortfall between the intended and actual participation of NGOs in ADB projects as well as their participation in these projects [47], recommending that before projects are approved, the ADB must make it “mandatory to include an agreed upon and verifiable monitoring system for NGO/CSO involvement in a project or program before it is approved” [8,47]. The study found “ADB’s capacity to mange and exchange information on the progress of NGO/CBO involvement and lessons learned from previous projects remains underdeveloped” [6,47].

Over the years, Japan, through its control over the Governing Board and the Presidency, along with the US, continued to have the greatest informal influence over policy decisions [45,48]. Together their economic policy ideologies, interests and strategies were learned, adopted, and implemented within the ADB [49], leading to the development of a multilateral agency that was accountable mainly to its largest financial contributors, rather than a diverse group of stakeholders representing a wide array of policy interests, ideologies, and beliefs.

By the 1990s, the governing structure of the ADB evolved along a predictable policy path, where, in line with path dependency theories of *power* concentration [27,31], ongoing financial and political investments by the largest shareholders helped strengthen their interests and policy influence [48]. Moreover, a path dependent instance of institutional *learning* occurred [19,31], whereby executive directors and especially staff members aligned themselves with Japan that was in charge of the Governing Board and the 12 Executive Directors [49]. This dominance limited the emergence of new policy ideas, such as to balance approaches fostering market based policies with those emphasizing poverty alleviation and equity in social welfare [42].

The challenges associated with these path dependency constraints, which emerged from the ADB’s originating coalitional politics, was a multilateral agency that was not fully capable of adapting to changing health environments and country needs, especially given ADB’s historically weak partnership and accountability to civil society. By the mid-1990s, the ADB attempted to rectify this problem through the creation of formal channels of communication [44], such as the creation of an “NGO Center” in 2000 [5,44], an “NGO Cooperation Network” in 2002 [5,44], a “Public Communications Policy” in 2003 [42], and the creation of an “Accountability Mechanism” that same year [50]. All of these representative institutions were created in order to learn from and work with NGOs, and were important for obtaining information about ongoing health and other social welfare needs. While some scholars consider the ADB as being transparent when compared to other multilateral agencies [51], others have argued that the ADB has not consistently engaged with civil society [42], limiting its ability to learn and adapt to new health conditions and citizens’ health needs, and that important information, such as policy divisions and partnerships with the private sector, have been withheld from civil society [5,42,51]. Despite the visible initiatives to work closely with civil society, the views and interests of civil society have not been formally adopted into the policy-making processes [5,41,44]. Instead, the major shareholders dominate the views emerging from the Governing Board [52,53].

Despite country needs for greater funding for public health [54], the ADB governing board does not appear to have prioritized health for investment [41,54]. Funding for health, nutrition, and social protection was second to last of the board’s investment categories in 2007, at US $95 million [41], an amount which does not reflect increased country needs for funding medical treatment, infrastructure, and health systems [41,42,48,49]. The unique political origins of the ADB, and the path dependent nature of the governing board’s structure appears to have reduced responsiveness to countries’ and citizens’ health needs [49,55,56], leading some scholars to argue for greater transparency, accountability, and equal participation within the ADB’s governing board [42,56]. But as Chavez [56] explains “there are just too many elements of the governance principle that threaten the very foundations of the Bank.”

### GAVI

The Global Alliance for Vaccines and Immunization was established in 2000. The originating interests and coalitions involved in forming GAVI differed from the ADB and the World Bank as they included a diverse body of actors, ranging from leaders of multilateral donor agencies (such as the World Bank, UNICEF, and WHO), philanthropic organizations, to the private sector, civil society, and academia [57-62].

In 1998, in response to a decline in international attention and financing for the vaccination of children in low-income countries, the World Bank President, James Wolfensohn, convened a summit in Washington D.C., bringing together the leaders of multilateral agencies, pharmaceutical companies, academia, and civil society, to explore how declining immunization levels could be addressed [58,60]. Concerned with the formation of yet another multilateral agency for health [57], summit members agreed to form an international partnership [60].

Shortly after the summit, philanthropist Bill Gates convened a special dinner that included the working group established by the Bank President James Wolfensohn, potential funders, activists, and representatives of NGOs. At this meeting the attendees explored innovative solutions and funding mechanisms to make available vaccines for children [60]. A second summit meeting was held in Bellagio, Italy, in 1999, bringing together key UN agencies, leaders of the vaccine industry, representatives of bilateral donor agencies, foundations, and civil society to create a new global partnership called the Global Alliance for Vaccines and Immunisation, with the remit to expand access to vaccines for children [58,60]. In November 1999, Bill Gates pledged US$780 million to the Fund for the Vaccination of Children, which fell under GAVI’s purview.

In 2000, at the World Economic Forum in Davos, Switzerland, the UN Secretary General Kofi Annan officially announced the establishment of GAVI that brought together public and private sector actors and interests [59,61], with a mission to guarantee and improve access to immunization for children; expand the use of existing cost-effective and safe vaccines; accelerate the development and introduction of new vaccines; and make immunization coverage an integral part of international development initiatives [59].

The diverse coalition of actors governing GAVI therefore shared similar policy interests and beliefs. First, all actors were interested in creating a diverse and inclusive governing body, which included individuals that could bring extensive knowledge and experience in providing immunization services. Second, all agreed that they would share their experience, talents, resources, and comparative advantages [62]. Third, they were unified in the belief that all children should have access to immunization and healthcare services [60,61]. Fourth, the coalition of actors believed that civil society should be an integral part of GAVI’s governance structure and delivery system [57,61,63,64].

Initially two boards of representatives governed GAVI. First, the Governing Board, which comprised four renewable representatives: the World Bank, WHO, UNICEF, and the Bill & Melinda Gates Foundation, and second, the Alliance Board, consisting of eleven rotating members, which includes several low- and high-income countries, such as Bhutan, Mali, Canada, the Netherlands and Norway, the pharmaceutical sector, the Rockefeller Foundation, research institutions, such as the US National Institutes of Health (NIH), and NGOs, such as Gates Children’s Vaccine Program and PATH [59].

In contrast to the BW institutions of the ADB and the World Bank, there was no single dominant actor within GAVI’s two governing boards. While a chairperson was elected for GAVI’s Governing Board, all participating actors agreed on an equal representation of board members and diversity of opinion [64], and also, that no country, constituency or organization should have control of the board. This inclusive arrangement has helped to ensure a diverse array of opinions and policy preferences. Although, others have argued that this governance arrangement has led to board member contestation over legislation [64], this arrangement has enabled all actors to contribute to GAVI’s governance and decision-making.

In further contrast to the BW institutions, civil society was involved in the formation of GAVI since its inception and had a permanent role on both governing boards, where the NGO representatives were *guaranteed* a seat, as their views and involvement were perceived to be critical in expanding immunization services to children in low-income countries [65,66]. NGOs also played an important role in country level implementation and governance, essentially being ‘the arms and ears of GAVI’. In contrast to the other board members, NGOs have provided a wealth of information on the design of programs, development of immunization policies, and effective ways of collaborating with local governments [61]. Since GAVI’s foundation, NGOs have been heavily involved in policy-making, holding governments accountable for their use of GAVI funding [61,65], increasing awareness of countries’ immunization challenges, as well as fund raising [66].

An inclusive board structure ensured a governance arrangement that was more responsive to a wider range of views, ideas, experiences, and interests with accountability to a wide array of stakeholders, [59,64] with no dominant board member(s) [64], despite the bulk of GAVI funding initially coming from the Gates Foundation and the United States, the United Kingdom, Japan, France, and the Netherlands [64]. Moreover, an inclusive and diverse board membership was maintained following the restructuring of GAVI boards from two to one [62], when civil society, NGOs and the private sector were able to maintain their one third overall representation on the new GAVI board [62].

In contrast to the ADB and the World Bank, GAVI’s diverse governance structure has enabled the organization to respond to emerging needs, priorities, and increased demand for vaccines by creating innovative funding mechanisms and strategies [57]. In response to requests from recipient countries, in 2009 GAVI’s board collaborated with the WHO, the World Bank, and the Global Fund to strengthen country health systems, especially human resources, and in developing supply chain management systems for vaccine distribution [57,62].

Since its inception, GAVI has made efforts to increase its accountability and responsiveness to civil society. This has been achieved through new policy initiatives and funding to ensure increased involvement of NGOs, not only for the implementation of immunization programs, but also on various policy committees, such as the Programme and Policy Committee and the Governance Committee, signaling to other board members that civil society is a vital partner for GAVI [57,61,62,65,67].

### The Global Fund

At the end of the 1990s, as the adverse health and economic consequences of the AIDS epidemic in Africa, Asia, and the Caribbean became evident, concerns grew worldwide on the negative impact of AIDS on development and global security. At the time, the lack of availability of a range of preventive and treatment interventions prompted activists and communities affected by AIDS to call for expanding access to life saving antiretroviral drugs to those in need as a human right. A vibrant and extensive global AIDS movement was born.

Suddenly, the AIDS epidemic and global health was at the nexus of economic development, human rights, and global security. Spurred by civil society, AIDS activists, the international health and development community, an international coalition of non-governmental organizations, bilateral donors, and global leaders formed to look for new ways to address AIDS, going beyond the existing international health, development, and financing agencies [68].

In 2000, AIDS and global health were discussed at the G8 meeting in Okinawa, Japan [68], with an acknowledgement of the need to provide large financial resources for AIDS. Soon after, the African Leaders Summit, held in April 2001 in Nigeria, called on donor countries as well as countries in Africa, through the Abuja Declaration, to make available finances to address HIV and AIDS in the continent [69]. At the summit, Kofi Annan, the United Nations Secretary General, called for the creation of a global fund to channel new funding to address the epidemic [68].

What happened next was unprecedented in the history of global health, a United Nations General Assembly Special Session on AIDS (UNGASS), held in June 2001 [70]. The UNGASS meeting was the first time the United Nations General Assembly had specifically focused on a disease, with an overwhelming support and commitment for a resolution to create a fund to address AIDS. Shortly after the UNGASS meeting, in July 2001, the G8 meeting in Genoa, Italy, endorsed the creation of a global fund with financing provided predominantly by the donor countries.

During this process, civil society, i.e., NGOs and activists, played an important role in bringing together the private sector, representatives of developed and developing countries, and other supportive organizations to help construct a global fund for combating AIDS [71,72]. These coalition-building efforts were facilitated by civil society’s drive to increase awareness, target new partners, and widely share information on funding and epidemiology [73]. NGOs and activists ensured broad media coverage of the event, raising the visibility of AIDS and the role of civil society in the fight against AIDS, as well as through targeted advocacy campaigns aimed at important decision-makers in government. Moreover, NGOs and activists worked assiduously to pressure governments and bilateral donors to finance a global response to AIDS [72].

Following the Genoa meeting, a multi-stakeholder Transitional Working Group, comprising bilateral donors, multilateral health and financing agencies, civil society, communities affected by AIDS and the private sector, was established and met in Brussels to develop a framework for the structure and operation of the Global Fund [71]. At this meeting, members of civil society, such as activists with a particular disease and NGO representatives, took the lead in creating a governing board membership structure that guaranteed civil society’s representation and participation in major funding decisions [72]. Civil society members were chosen regardless of their personal experiences, policy views, and interests. During this period, civil society representatives succeeded in stressing the need for their inclusiveness in policy decision-making, as well as a balanced, fair, and transparent discussion of funding priorities and policies [73].

The deliberations of the Transitional Working Group led to the development of a Framework Document [13], which identified the set of principles on the governance, structure, focus and operations of the Global Fund. These principles guide the Global Fund to: (i) Operate as a financial instrument, not an implementing entity; (ii) make available and leverage additional financial resources; (iii) support programs that evolve from national plans and priorities; (iv) operate in a balanced manner in terms of different regions, diseases and interventions; (v) pursue an integrated and balanced approach to prevention and treatment; evaluate proposals through independent review processes, and (vi) operate with transparency and accountability [74]. The principle of supporting programs that evolve from country national plans and strategies underpins country ownership and the active participation of civil society.

Indeed one of the key normative claims underpinning the Framework Document is the Global Fund Board’s belief and commitment to fully representing the views, experiences, and needs of civil society [13]. It is a belief that stems from the realization that in order to create policies that effectively represent the views and needs of those affected by HIV/AIDS, tuberculosis and Malaria, civil society’s participation is equally if not more legitimate and helpful than representatives from government and/or the private sector [72,73]. Indeed Governing Board decisions balance civil society needs with those of the government and/or private sector [72]. This approach breaks from the “business as usual” [72] model where representatives of nation states have historically designed and implemented policy. The Global Fund Framework Document, therefore, has helped civil societal representation and strengthened governance processes [72,73].

Thanks in part to civil society’s stewardship in creating the Global Fund, its governance structure was characterized by broad multi-stakeholder involvement at all levels, the Board, the Committees of the Board, and the Country Coordinating Mechanisms (CCMs) at country level [13]. The Board is diverse in its nature and consists of 20 voting members and eight non-voting members [13]. All Board Members represent “constituencies” comprising “a group of communities, networks, governments or institutions”, namely:

(i) ‘Donor Countries’ that provide financial contributions to the Global Fund and have 8 seats;

(ii) ‘Implementing Countries’, that have seven seats and represent the low and middle-income countries benefiting from Global Fund financing and which are grouped according to the six World Health Organization regions, plus an additional seat for Africa;

(iii) ‘Communities’ (one seat) affected by AIDS, tuberculosis and malaria;

(iv) ‘Civil Society from Developing Countries’ (one seat) that consist of NGOs that are based in implementing countries, including faith-based organizations, health service providers, advocacy groups and professional associations, coordinated by the international Council of AIDS Service Organizations (ICASO);

(v) ‘Civil Society from Developed Countries’ (one seat) that include NGOs based in Western Europe, the United States, Canada, Japan, Australia and New Zealand, as well as international nongovernmental organizations with headquarters in developed countries;

(vi) ‘Private Sector’ (one seat) coordinated by the Global Business Council on HIV/AIDS, Tuberculosis and Malaria, and;

(vii) ‘Foundations’ (one seat) which provide financial and resource support to the Fund;

Non-voting constituencies that collectively have eight seats and consist of the ‘Partners’ constituency (which includes Roll Back Malaria Partnership, Stop TB Partnership and UNITAID), the United Nations constituency (which includes the United Nations Joint Programme on HIV/AIDS [UNAIDS] and the World Health Organization), as well as the World Bank, which also acts as the Trustee of the Global Fund [75].

In addition to the Board, Committees and the CCMs, the Global Fund also incorporates a Partnership Forum as part of its governance structure. The Partnership Forum, which meets once every 18 months to two years, is inclusive, bringing together a broad range of stakeholders that includes among others grant recipients, civil society, NGOs, affected communities, donors, international institutions, technical agencies, research organizations, academic community, foundations, and private business. As set in the Global Fund by-laws, the Partnership Forum “provides an important and visible platform for debate, advocacy, continued fundraising, and inclusion of new partners.” It is an important “communication channel for those stakeholders who are not formally represented elsewhere in the governance structure.” The Partnership Forum reviews progress based on reports from the Board, provides input to Global Fund strategy and helps set its direction [76]. The Partnership Forum provides the opportunity for the governing board to understand the needs of civil society and respond to their requests [72]. For example, in 2006 in Durban more than 414 people, comprising activists, NGOs, and private sector representatives from 118 countries, met with Global Fund board members [77]; the goal of this Partnership Forum meeting was for the board to obtain feedback from Global Fund operations, learn about medical and prevention needs, to respond and incorporate society’s policy recommendations, which ultimately led to the Global Fund Strategy report of 2007 [77].

The CCMs are multi-sectoral institutions that bring together representatives of governments, bilateral donors, multilateral agencies, civil society, NGOs, faith-based institutions, academic institutions, private sector, and communities affected by AIDS, tuberculosis and malaria. The CCMs coordinate the development and submission of proposals for funding requests to the Global Fund and oversee grant implementation.

Over the years, the creation of a multi-sectoral governing board within the Global Fund has led to a board that is accountable and responsive to the needs of civil society and governments. Because NGOs, individuals afflicted by particular diseases, and the private sector are guaranteed a voice on the board, and because there is a very strong connection between civil society and transnational activist groups [77,78], the board responds to and incorporates requests on ways to improve its policy-making and financial assistance to grant recipients. The Global Fund board has responded to country needs for financing health systems responses for AIDS, TB, and malaria, mainly through enhanced supply chains for antiretroviral medications, human resources, and training [79]. A study by the International Center on Research for Woman found that the board and executive management demonstrated a high level of commitment to responding to civil society’s recommendations for how to improve funding for medical treatment, prevention activities (especially in the area of gender and human rights), while highlighting the ease with which civil society board members could approach the Chair and Executive Director and the latter’s willingness to hear and respond to their concerns [80]. The study noted that following representations by activists and the NGO representatives on the Board for the full disclosure of the working policy documents of the board, the Chair of the board and other board members responded by adopting the *Global Fund Documents Policy*, which details 10 categories of documents that are routinely made public [80].

In 2003 the Global Fund Board created a “Civil Society Team” within the Global Fund’s External Relations Unit; this Team frequently met with activist and NGOs in Geneva or in recipient countries in order to learn about their needs for accessing drugs and providing prevention services while discussing and incorporating their recommendations during board meetings [47]. Further, the Board has created various forums in which NGOs are encouraged to participate, to share their views, and where Global Fund board members and staff can learn about needs and incorporate them into policy decisions [47]. All of these efforts have convinced analysts that the Global Fund’s governing board is indeed committed to being both responsive and accountable to civil society, learning from them and incorporating their views [47,80].

The strong involvement of civil society in the creation of the Global Fund has meant that governance arrangements have guarded the interests of civil society, affected communities, and implementing countries. Although the accountability of the Board and senior officials to taxpayers who provide almost 95% of Global Fund financing is not necessarily clear, the organization’s responsiveness to civil society is a marked break from the BW institutions already discussed.

## Conclusion

The rich diversity of political and civil societal interests that shape the formation of domestic political institutions are also present during the formation of multilateral donor agencies in global health. Multilateral agencies committed to helping nations overcome health challenges exhibit vast differences in their political and civic origins. These origins not only provide us with a more accurate indication of the type of multilateral agencies that they are, e.g., Bretton Woods versus proto-institutions, but they also provide insights into why and how agencies behave differently. By focusing on multilateral donor agencies’ strengthened governance processes, our analytical approach has highlighted considerable variation in these outcomes between these different types of multilateral donors in global health.

The Global Fund and GAVI, for example, were more responsive to civil society’s health needs, which included obtaining medications for essential medicines, healthcare treatment, as well as information for awareness and prevention, and were more willing to institutionalize their interests early on. These needs are often the product of objective assessments of drug shortages and funding for prevention and treatment conducted by activists affected by these diseases, as well those NGOs that represent them. But it is important to note that not all activists and NGOs are successful in increasing awareness, communicating needs, and mobilizing a response to disease. In Brazil, for example, while activists and NGOs have succeeded in achieving their objectives for AIDS, less funding, volunteer experience, and the stigma attached to tuberculosis as a disease of the poor, tuberculosis activists have been less successful [81,82]. Future research will need to compare and explain why some health sectors are more successful than others in communicating their needs, mobilizing, and thus providing more effective representation within multilateral health agencies.

In contrast, while the BW institutions of the World Bank and the Asian Development Bank also expressed commitments in achieving these outcomes, they experienced challenges in implementing reforms to achieve greater responsiveness and accountability to civil society. We have argued that differences in these strengthened governance processes reflect differences in the political and civil societal factors leading to the formation of these multilateral agencies. When these agencies were crafted by political elites from advanced industrialized nations, initial discussions, coalitions, and governance boards were shaped by those same elites; over time, their interests superseded competing interests, which sought to develop more accountable institutions. Conversely, when multilateral agencies were initiated and created by civil society, as well as other non-governmental sectors, such as the private sector, this led to the formation of coalitions and governing boards that were more representative to civil societal needs. These proto-institutions, i.e., the Global Fund and GAVI, were more capable in adapting, responding to, and incorporating the needs of not only civil society, but other emerging actors as well, such as affected communities, philanthropic agencies, and private sector institutions that play an important role in the global health landscape.

In addition, a key takeaway message emerges from our study: that is, because of the diversity and early coalition building processes that characterized the establishment of the Global Fund and GAVI, the governing boards of these proto institutions were able to avoid the path dependency problems experienced by the established multilateral agencies of the World Bank and the ADB with origins in Bretton Woods. It therefore seems that in the future, the only agencies that will be capable of avoiding these path dependency problems are those that were recently created and/or new agencies established by a diverse array of state and non-state actors. Our approach therefore helps to explain why not only the World Bank and ADB, but other older BW institutions, such as the WHO, have had a difficult time strengthening its governance processes [83].

As the factors that determine the accountability and responsiveness of multilateral donor agencies depend on their originating coalitional politics, we suggest an approach that combines the literature addressing the non-institutional political origins of institutions, proto-institutions, and path dependency theory. A particular advantage with our proposed origins and proto-institutional approach is that it helps to explain the actors, interests, and coalitions leading to the formation of governance arrangements within multilateral agencies, the board structure, rules, and accountability processes. Path dependency theory also helps to provide insight into the board’s subsequent policy beliefs, interests, and reform strategies, and to what extent self-reinforcing mechanisms such as *increasing returns* and *legitimacy* create disincentives to purse reform. This was the case with the BW institutions of the World Bank and ADB but not GAVI and the Global Fund. When combined, we have argued that a framework accounting for the origins and subsequent institutional logic of multilateral agencies helps to more accurately describe the types of multilateral donors that they are, as well as helping to explain their capacity to adapt to ongoing country and civil societal healthcare needs. Through our framework, moreover, we also found that BW institutions have not been as effective as the more recently established proto-institutions in achieving strengthened governance processes.

But there are also policy implications associated with these strengthened governance processes. First, by achieving this outcome, funding policies may be designed more effectively to meet citizens and countries’ healthcare needs. This is because increased representation, accountability, and responsiveness helps to ensure that governing boards have the most accurate, up to date information that they need in order to make sound policy decisions. And second, strengthening transparency and accountability to civil society increases the likelihood that funding will be used effectively and for the right purposes.

While we have suggested a new approach to explaining differences between the strengthened governance processes of multilateral donor agencies, we have not addressed the differences *within* BW and proto-institutions. With respect to similar types of BW institutions, scholars will need to examine to what extent and how they vary in terms of governing boards’ willingness and commitment to become more accountable, responsive, and inclusive of civil societal needs, and the factors that account for these differences.

The global economic recession and reduced contributions to multilateral funders of global health may lead to *de-legitimization* of existing policy views and traditions in governance and accountability and precipitate institutional *conversion*[83-86]. Perhaps the need for greater efficiency when targeting and using multilateral funds will force governing boards to restructure themselves and become more open, accountable, and inclusive of civil societal recommendations. Scholars will need to examine differences in the impact of exogenous economic conditions, and how BW governing boards are responding differently to these new environments. It may certainly be the case that some BW institutions, such as the World Bank, will become more accountable and responsive, as compared with other BW institutions, such as the ADB and/or the African Development Bank, whose financial budget and funding contributions, as well as global attention and responsibility, are comparatively smaller.

Finally, scholars may seek to establish differences between similar types of proto-institutions. To what extent, for example, has the new economic climate and a decrease in financial contributions to the Global Fund led to subtle yet fundamental differences in governing board accountability and responsiveness to civil societal needs? Furthermore, in a context of increased need for global health funding, has the rise in influence of philanthropic organizations, such as the Bill & Melinda Gates Foundation and the Rockefeller Foundation, strengthened the influence of NGOs and community-based organizations which they fund, and has this, in turn, increased civil society’s influence within the Global Fund and GAVI? Have these private donors become more influential within the Global Fund and GAVI and how has this influenced donor priorities? Future researchers will need to address these questions, closely examining the external factors that may lead to differences in proto-institutional accountability and responsiveness in the new climate of economic uncertainty.

## Endnotes

^a^ Note, however, that agencies need not address all of these aspects of health governance in order to strengthen them. As the case studies of the World Bank, ADB, the Global Fund, and GAVI will illustrate, these agencies varied in terms of the aspects of health governance that they addressed.

^b^ In our causal argument, we certainly acknowledge the presence of causal endogeneity. This is because individuals having participated in creating governance structures in these agencies often eventually sit on the very governing boards that they created, possessing the same policy interests and incentives; these board members in turn affect subsequent reform coalition processes and governance outcomes. We find endogeneity to be permissible if the goal is to explain the rise of a particular institution (e.g., agency), which is the case in our approach; conversely, it is unsuitable if our interest is in establishing independent casual variables and outcomes.

^c^ It is important to note, however, that the BW institutions have many more health and social welfare sectors to govern – approximately 19 – when compared to the proto-institutions involved in global health. This excessive amount of responsibility may pose greater challenges for strengthening governance processes in the BW versus the proto-institutions. Future research will need to compare and assess governance processes within different types of health and social welfare sectors within the BW institutions and to see if this complexity is hampering efforts to strengthen governance processes.

## Abbreviations

Global Fund: Global Fund to Fight AIDS, Tuberculosis and Malaria; GAVI: Global Alliance for Vaccines Initiative; NGO: Non-governmental organization; BW: Bretton Woods; ADB: Asian Development Bank; IDB: Inter-American Development Bank; IBRD: International Bank for Reconstruction and Development; IFC: International Financial Corporation; MIGA: Multilateral Investment Guarantee Agency; CAO: Compliance Advisor/Ombudsman Office; ECAFE: Economic Commission for Asia and the Far East; CCM: Country coordinating mechanisms.

## Competing interests

The authors declare that they have no competing interests.

## Authors’ contributions

EG and RA carried out the research and writing for this article; M McCaffrey provided research assistance. All authors read and approved the final manuscript.
